# Long-Term Dementia Risk in Parkinson Disease

**DOI:** 10.1212/WNL.0000000000209699

**Published:** 2024-08-07

**Authors:** Julia Gallagher, Caroline Gochanour, Chelsea Caspell-Garcia, Roseanne D. Dobkin, Dag Aarsland, Roy N. Alcalay, Matthew J. Barrett, Lana Chahine, Alice S. Chen-Plotkin, Christopher S. Coffey, Nabila Dahodwala, Jamie L. Eberling, Alberto J. Espay, James B. Leverenz, Irene Litvan, Eugenia Mamikonyan, James Morley, Irene H. Richard, Liana Rosenthal, Andrew D. Siderowf, Tatyana Simuni, Michele K. York, Allison W. Willis, Sharon X. Xie, Daniel Weintraub, for the Parkinson's Progression Markers Initiative

**Affiliations:** From the Departments of Neurology (J.G., A.S.C.-P., N.D., J.M., A.D.S., A.W.W., D.W.) and Psychiatry (E.M., D.W.), and Biostatistics and Epidemiology (S.X.X.), University of Pennsylvania, Philadelphia; Department of Biostatistics (C.G., C.C.-G., C.S.C.), University of Iowa; Department of Psychiatry (R.D.D.), Rutgers University, Newark, NJ; Department of Old Age Psychiatry (D.A.), Kings College London, UK; Neurological Institute (R.N.A.), Tel Aviv Sourasky Medical Center, Israel; Department of Neurology (R.N.A.), Columbia University Irving Medical Center, New York, NY; Department of Neurology (M.J.B.), Virginia Commonwealth University, Richmond, VA; Department of Neurology (L.C.), University of Pittsburgh, PA; The Michael J. Fox Foundation for Parkinson's Research (J.L.E.), New York, NY; James J. and Joan A. Gardner Family Center for Parkinson's Disease and Movement Disorders (A.J.E.), Department of Neurology, University of Cincinnati; Cleveland Clinic (J.B.L.), Neurological Institute, Lou Ruvo Center for Brain Health, OH; Department of Neuroscience (I.L.), University of California San Diego; Parkinson's Disease Research, Education and Clinical Center (PADRECC) (J.M., D.W.), Crescenz Veteran's Affairs Medical Center, Philadelphia, PA; Department of Neurology (I.H.R.), University of Rochester, NY; Department of Neurology (L.R.), Johns Hopkins University, Baltimore, MD; and Department of Neurology (T.S.), Northwestern University, Chicago, IL; Departments of Neurology and Psychiatry and Behavioral Sciences (M.K.Y.), Baylor College of Medicine, Houston, TX.

## Abstract

**Background and Objectives:**

It is widely cited that dementia occurs in up to 80% of patients with Parkinson disease (PD), but studies reporting such high rates were published over two decades ago, had relatively small samples, and had other limitations. We aimed to determine long-term dementia risk in PD using data from two large, ongoing, prospective, observational studies.

**Methods:**

Participants from the Parkinson's Progression Markers Initiative (PPMI), a multisite international study, and a long-standing PD research cohort at the University of Pennsylvania (Penn), a single site study at a tertiary movement disorders center, were recruited. PPMI enrolled de novo, untreated PD participants and Penn a convenience cohort from a large clinical center. For PPMI, a cognitive battery is administered annually, and a site investigator makes a cognitive diagnosis. At Penn, a comprehensive cognitive battery is administered either annually or biennially, and a cognitive diagnosis is made by expert consensus. Interval-censored survival curves were fit for time from PD diagnosis to stable dementia diagnosis for each cohort, using cognitive diagnosis of dementia as the primary end point and Montreal Cognitive Assessment (MoCA) score <21 and Movement Disorder Society—Unified Parkinson's Disease Rating Scale (MDS-UPDRS) Part I cognition score ≥3 as secondary end points for PPMI. In addition, estimated dementia probability by PD disease duration was tabulated for each study and end point.

**Results:**

For the PPMI cohort, 417 participants with PD (mean age 61.6 years, 65% male) were followed, with an estimated probability of dementia at year 10 disease duration of 9% (site investigator diagnosis), 15% (MoCA), or 12% (MDS-UPDRS Part I cognition). For the Penn cohort, 389 participants with PD (mean age 69.3 years, 67% male) were followed, with 184 participants (47% of cohort) eventually diagnosed with dementia. The interval-censored curve for the Penn cohort had a median time to dementia of 15 years (95% CI 13–15); the estimated probability of dementia was 27% at 10 years of disease duration, 50% at 15 years, and 74% at 20 years.

**Discussion:**

Results from two large, prospective studies suggest that dementia in PD occurs less frequently, or later in the disease course, than previous research studies have reported.

## Introduction

Cognitive decline is a major nonmotor manifestation in Parkinson disease (PD), and development of dementia (PDD) is a feared long-term outcome. Initial changes in cognition can occur before PD diagnosis,^1-5^ and 10%–20% of patients with newly diagnosed PD have cognitive deficits.^6-8^ In addition, approximately 25% of non-demented patients have mild cognitive impairment (MCI),^9^ which predicts faster conversion to dementia.^10,11^ Thus, cognitive changes can occur throughout the disease course and increase in severity and prevalence over time.

Initial cognitive impairments in PD can occur in a range of cognitive domains.^9,12,13^ Predictors of cognitive impairment include older age and age at disease onset, greater disease severity, postural instability-gait disorder subtype, REM sleep behavior disorder, and psychiatric symptoms (e.g., psychosis and depression).^14^

Unfortunately, treatment of PD cognitive impairment lags treatment of motor symptoms. To optimize clinical management, better understand progression to dementia, and spur treatment development, accurate estimates of long-term dementia rates are needed. Yet, epidemiologic research into long-term cognitive outcomes has been limited.

It is commonly cited that dementia occurs in at least 80% of patients with PD in the long term. An early regional population-based study in Norway recruited 224 patients between 1992 and 1993 with established PD at baseline, 87 of whom were also evaluated at year 8 (2000–2001); this study reported a 78% cumulative dementia rate.^15^ Another early clinic-based study in Australia recruited 136 patients with PD between 1984 and 1986 at disease onset with 30 patients assessed after 20 years (2004–2007), 83% of whom had dementia.^16^ Although these studies have been important for the field in underlining the importance of cognitive decline in PD, both studies are over 15 years old, had relatively small sample sizes, lacked enrollment criteria, had high age at enrollment, and either used limited cognitive testing or did not have a dementia diagnosis made by a study investigator.

Other regional population-based studies have followed participants from disease onset. The CamPaIGN study followed 142 patients from diagnosis in 2000–2002, with 49 patients remaining at the 10-year follow-up (2012); the dementia rate was 46%.^17^ Another study found that 46% (93/201) of patients, recruited in two phases from 2002 to 2004 and 2006 to 2009, had developed dementia after a median of 10 years of disease duration and estimated a dementia rate of 80% by year 15.^18^ Finally, a study that began recruitment in 2009 with a 10-year follow-up reported an estimated dementia rate of 54% (64/143) among patients with newly diagnosed PD.^19^

There are also larger scale, national population-based PD dementia outcome studies, suggesting lower long-term rates of dementia, but with unclear results. For example, 2 Taiwanese studies reported dementia rates out to 11 years disease duration, based on a medical record diagnosis. The first study that retrospectively recruited participants from 2002 to 2003 and followed up to 2012 reported a dementia rate for survivors at year 11 to be 39% (20) while the other recruited from 2001 to 2005 and followed to 2011 reported 12% (21), despite examining the same population. In Sweden, a population-based sample from 2011 to 2012 found the incidence rate of dementia to be 50%–60% (683/1,369) after 25 years of disease duration.^20^

Given the conflicting data, we aimed to re-evaluate the long-term dementia risk in PD by analyzing data from two rigorous, large, longitudinal, prospective, observational studies, the Parkinson's Progression Markers Initiative (PPMI) and a NIH-funded study at the University of Pennsylvania (Penn).

## Methods

### PPMI Cohort

The PPMI study and cohort has been extensively described.^21,22^ For the original cohorts, participants with PD and healthy controls (HCs) of similar age and sex were recruited from 24 study sites in the United States (18), Europe (5), and Australia (1). Both participants with PD and HCs were recruited through local events, extensive international publicity, and physician referrals.^21^ At each annual study visit, the local site investigators reassessed the subject diagnosis to identify any non-PD participants. At enrollment, participants with PD were required to be 30 years or older, untreated, within 2 years of diagnosis, and Hoehn and Yahr stage <3 and to have either at least two of resting tremor, bradykinesia, or rigidity (must have either resting tremor or bradykinesia), or a single asymmetric resting tremor or asymmetric bradykinesia. All participants with PD underwent dopamine transporter imaging with 123I ioflupane or vesicular monoamine transporter imaging and were eligible only if either demonstrated dopaminergic deficit consistent with PD. HCs were required to be 30 years or older without an active, clinically significant neurologic disorder or a first-degree relative with PD and have a Montreal Cognitive Assessment (MoCA)^23^ score ≥27. Data collection occurred between 2010 and 2013.

Assignment of a cognitive diagnosis (normal cognition, MCI, or dementia) is made at each visit by the site investigator, which was not fully implemented until study year 3.^24^ The site investigator is provided a guidance document on how to assess for subjective cognitive change compared with pre-PD state, impairment in cognitive abilities, and functional impairment due to cognitive deficits (i.e., providing specific examples of how cognitive impairment might adversely affect instrumental activities of daily living requiring cognitive abilities), with the option to review cognitive test results (e.g., MoCA, Hopkins Verbal Learning Test-Revised,^25^ Benton Judgment of Line Orientation,^26^ Symbol-Digit Modalities Test,^27^ Letter-Number Sequencing,^28^ and category [animal] fluency^29^) (eAppendix 1). The guidance document was meant to approximate PD-MCI^30^ or PDD^31^ criteria.

Progression to dementia between participants with PD and HCs was also compared. For this analysis, we used time since enrollment rather than time since diagnosis because HCs do not have the latter. Ultimately, there were not an adequate number of participants who developed dementia over time to allow subgroup analyses.

### Penn Cohort

The Penn cohort and cognitive consensus process has also been described.^32^ A convenience sample of patients with clinically diagnosed^33^ PD receiving care at a tertiary movement disorders center was recruited from 2006 to 2021 for a long-term observational study focused on predictors of disease progression, including cognition. Participants were either approached for enrollment or referred by their clinician. Visits are conducted annually for the first four years and then biennially until completion. At each visit, participants are administered a detailed cognitive battery, including global tests (i.e., MoCA and Dementia Rating Scale-2^34^) and eight other domain-specific cognitive tests. For each visit, experts review cognitive test results, questionnaires assessing cognitive function, and clinician impression of cognitive status and then assign a diagnosis of normal cognition, MCI, or dementia consistent with PD-MCI^30^ and PDD^31^ criteria (eAppendix 2).

To determine PD duration, participants provided their closest estimate of diagnosis date as the exact date, month/year (set to the 15th of the month), or calendar year (set to July 1 of year). If necessary, a participant's electronic medical record was accessed to verify year of disease diagnosis. All participants were required to have a minimum of two visits to be included, unless demented at the baseline study visit.

### Analyses

Analyses and output were generated using SAS version 9.4 (SAS/STAT 15.1; SAS Institute Inc., Cary, NC). Nonparametric survival plots using the expectation-maximization iterative convex minorant algorithm using the ICLIFETEST procedure in SAS with imputed standard error for interval-censored data were generated for time from PD diagnosis (time of symptom onset unavailable) to dementia diagnosis for each cohort. Pointwise confidence limits of the survival function were obtained using a log-log transformation. Additional survival curves were fit for the PPMI cohort using dementia proxy end points: MoCA score <21^35^ and Movement Disorder Society—Unified Parkinson's Disease Rating Scale (MDS-UPDRS) 1 cognitive score ≥3 (moderate or severe). The MDS-UPDRS Part I cognitive score of 3 states that “cognitive deficits interfere with but do not preclude the patient's ability to carry out normal activities and social interaction”; this description of functional impairment aligns with the diagnostic criteria for a PD dementia diagnosis^31^ and thus was chosen as a secondary end point. The inclusion of the MDS-UPDRS Part I cognition and MoCA cutoff scores was meant to provide complementary ways of assessing dementia level cognitive impairment to determine whether the results were consistent with site investigator diagnosis. Time was calculated as the years from PD diagnosis to a stable dementia diagnosis, with intervals defined as time between the first instance of a stable dementia diagnosis (i.e., dementia diagnosis at all subsequent visits) and the prior visit. If Penn participants had a consensus diagnosis of dementia at the baseline study visit, we assumed that the dementia occurred in the interval between PD diagnosis and the baseline visit (i.e., the left bound of the interval was time 0 and right bound was the time from PD diagnosis to baseline visit). Participants who did not receive a dementia diagnosis, achieve a stable diagnosis, or were lost to follow-up were right censored. Time to censoring was calculated as the number of years from the date of PD diagnosis to their last follow-up date. In addition, cumulative dementia diagnoses and probability of dementia by year of disease duration were tabulated for both cohorts (out to 10 years for PPMI and 35 years for Penn).

For PPMI, 16 participants were removed from the primary analysis because of missing investigator cognitive diagnosis data at all visits. To address informative missingness in this cohort, a worst-case sensitivity analysis was performed by imputing participants as having a dementia diagnosis at the next interval visit if they dropped out before receiving a dementia diagnosis or completing the study. For the comparison of participants with PD and HCs in the PPMI cohort, participants with PD were divided into those with a MoCA score ≥27 (the same as the inclusion criterion for HCs) and those with a score of <27 at the baseline study visit.

For Penn, cohort subanalyses were stratified by sex, age at PD diagnosis, and years of formal education. The estimated occurrence of dementia diagnoses was compared between groups using a generalized log-rank test.

### Data Availability

PPMI data used in the preparation of this article were obtained on February 27, 2023, from the PPMI database (ppmi-info.org/access-data-specimens/download-data; RRID:SCR 006431). Study protocol and manuals are available at ppmi-info.org/study-design. Penn data were downloaded from the Penn Integrated Neurodegenerative Disease database on March 6, 2023. The Penn cohort protocol and deidentified data may be shared on request by qualified investigators for the purposes of replicating procedures and results (contact the corresponding author).

## Results

### Cohort Descriptions

#### PPMI Cohort

Of the original 423 de novo, untreated-at-baseline PD participants, 6 were rediagnosed, leaving 417 with a diagnosis of idiopathic PD. The cohort included sporadic PD (397, 95%), PD with *GBA1* pathogenic sequence variants (13, 3%), PD with *LRRK2* G2019S variation (6, 1%), and PD with *GBA1* + *LRRK2* mutation (1, 0.2%). Demographic and clinical characteristics for the PPMI cohort are presented in the Table.

**Table:** Demographic and Clinical Characteristics at the Baseline Visit

Variable	Cohort	
	PPMI cohort (N = 417)	Penn cohort (N = 389)
Age at baseline visit, y		
Mean (SD)	61.6 (9.8)	69.3 (8.0)
Min, max	33, 85	49, 94
PD duration at baseline visit, y		
Mean (SD)	0.6 (0.5)	6.3 (5.3)
Min, max	0, 3	0, 32
Age at PD diagnosis categories, n (%)		
Age <56	138 (33)	89 (23)
Age 56–70	212 (51)	213 (55)
Age >70	59 (14)	87 (22)
Missing	8 (2)	0
Sex, n (%)		
Male	272 (65)	261 (67)
Female	145 (35)	128 (33)
Race, n (%)		
White	385 (92)	362 (93)
Non-White	29 (7)	27 (7)
Missing	3 (1)	0
Education, y		
Mean (SD)	15.6 (3.0)	16.0 (2.5)
Min, max	5, 26	8, 21
MDS-UPDRS III total score		
Mean (SD)	20.9 (8.9)	28.2 (13.2)^a^
Min, max	4, 51	2, 85
Missing	0	6
Hoehn and Yahr, n (%)		
1	183 (44)	33 (8)
2	232 (56)	151 (39)
3	2 (<1)	185 (48)
4/5	0	16 (4)
Missing	0	4 (1)
MoCA		
Median	28	26
Min, max	17, 30	7, 30
Missing	0	215^b^
LEDD		
Median	NA	600
Min, max	NA	0, 2,960

Abbreviations: LEDD = levodopa equivalent daily dose; MDS-UPDRS III = Movement Disorder Society—Unified Parkinson's Disease Rating Scale part III; MoCA = Montreal Cognitive Assessment; NA = not available; PD = Parkinson disease; Penn = University of Pennsylvania; PPMI = Parkinson's Progression Markers Initiative.

a UPDRS III score converted to MDS-UPDRS III score for the Penn cohort.^41^

b MoCA score not administered at the baseline visit for the Penn cohort for original participants.

For the primary analysis (N = 401 with site investigator cognitive diagnosis), longitudinally followed participants were categorized as active (N = 199, 49.6%), completed (i.e., did not reconsent for ongoing participation after the initial 5-year study period; N = 23, 5.7%), or withdrawn/dropout (N = 145, 36.2%) or were endpointed with a dementia diagnosis (N = 34, 8.5%). Among dropouts, 55 instances were because of withdrawn consent, 29 lost to follow-up, 28 died, and 33 for other reasons.

#### Penn Cohort

The Penn cohort included 389 participants with PD with the requisite data followed over time, with mean (range) PD duration 6.3 (0–32) years at the baseline study visit and mean number of follow-up visits (1–2 years apart) 4.6.

Participants were considered active (N = 106, 27.2%), deceased (N = 46, 11.8%), withdrawn/dropout (N = 53, 13.6%), or demented (N = 184, 47.3%). Of those who dropped out, 15 moved, 14 requested to be withdrawn, 14 lost contact, 5 were outside the visit window, and 5 were too physically disabled. Demographic and clinical characteristics for the Penn cohort are also presented in the Table.

### Long-Term Dementia Outcomes in De Novo PD: PPMI Cohort

No participants in PPMI were diagnosed with dementia at the baseline study visit; 28 of 401 (7.0%) were diagnosed with dementia by the site investigator by year 10; 34 (8.5%) were diagnosed with dementia over the entire follow-up period. When using alternate definitions for dementia, 41 of 417 (9.8%) participants had dementia defined by MoCA cutoff by year 10 and 49 (11.8%) had dementia overall; 31 of 417 (7.4%) participants had dementia defined by the MDS-UPDRS I cognition score by year 10 and 47 (11.3%) had dementia overall.

**Figure 1:**
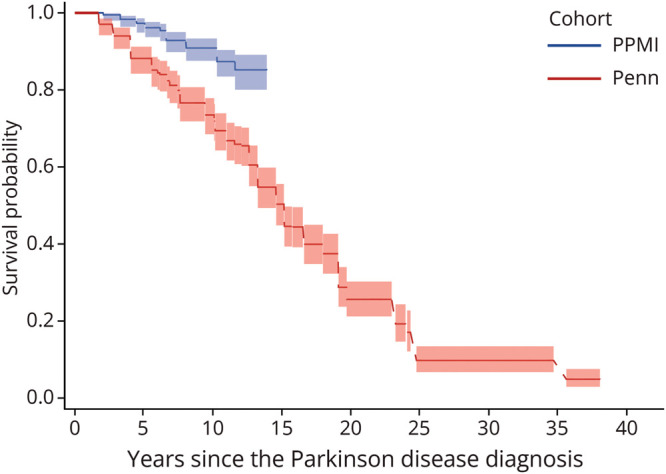
Time From PD Diagnosis to Site Investigator Dementia Diagnosis in PPMI and Penn Cohorts PD = Parkinson disease; Penn = University of Pennsylvania; PPMI = Parkinson's Progression Markers Initiative.

Survival curves for PPMI are displayed in Figure 1 and eFigures 1 and 2. The median time to dementia diagnosis from PD diagnosis could not be estimated because the cumulative probability of dementia over the entire follow-up period never reached more than 50%. The estimated probability of dementia and pointwise confidence intervals are presented in eTable 1.

### Long-Term Dementia Outcomes in Established PD: Penn Cohort

Of the 389 participants, 42 (10.8%) were diagnosed with dementia at the baseline study visit and 184 (47.3%) were diagnosed with dementia through the consensus process at some point. The survival curve is presented in Figure 1. The median time to dementia diagnosis from PD diagnosis was 15.2 years (95% CI 13.3–15.2). The estimated probability of dementia and pointwise confidence intervals are presented in eTable 2.

### Dementia Outcomes by Age at Disease Diagnosis, Sex, and Education in Penn Cohort

**Figure 2:**
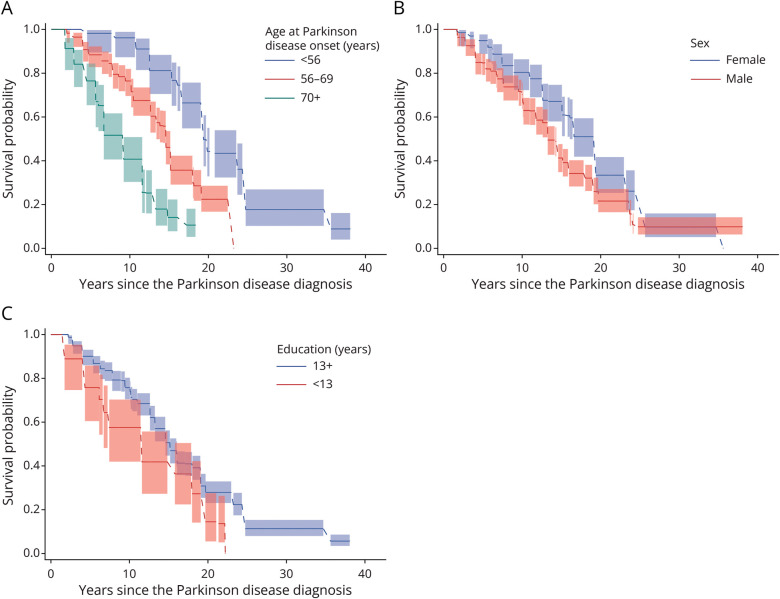
Estimated Times From PD Diagnosis to Dementia Diagnosis Estimated times (A) by age at PD diagnosis in the Penn cohort, (B) by sex in the Penn cohort, and (C) by education level in the Penn cohort (with 95% pointwise confidence limits). PD = Parkinson disease; Penn = University of Pennsylvania.

By age at disease diagnosis, 36 (40.4%) among those who were younger than 56 years, 95 (44.6%) among those aged 56–70 years, and 53 (60.9%) among those older than 70 years were diagnosed with dementia. The median time to dementia diagnosis from PD diagnosis was 19.4 (95% CI 19.4–23.7) years among those who were younger than 56 years, 14.6 (95% CI 13.4–15.2) years among those aged 56–70 years old, and 9.2 (95% CI 6.7–11.6) years among those older than 70 years (Figure 2A). These differences in time to dementia differed significantly by age at PD diagnosis groupings (generalized log-rank test *p* < 0.001). The estimated probability of dementia and cumulative dementia diagnoses by age at PD diagnosis are presented in eTable 3.

For analyses of outcomes by sex, 49 (38.3%) female and 135 (51.7%) male participants were diagnosed with dementia. The median time to dementia diagnosis from PD diagnosis was 19.4 (95% CI 16.1–19.4) years for female participants and 13.3 (95% CI 13.3–14.6) years for male participants (Figure 2B), a significant difference (generalized log-rank test *p* = 0.004). The estimated probability of dementia and cumulative dementia diagnoses by sex are presented in eTable 4.

When examining education, 30 (58.8%) of those with <13 years and 154 (45.6%) of those with ≥13 years of education were diagnosed with dementia. The median time to dementia from PD diagnosis among those with <13 years of education was 11.6 (95% CI 6.7–18.0) years and for participants with ≥13 years of education was 15.2 (95% CI 14.6–16.1) years (Figure 2C). The difference in time to dementia was significantly different (*p* = 0.006). The estimated probability of dementia and cumulative dementia diagnoses by education level are presented in eTable 5.

### Comparison of Participants With PD and HCs in PPMI Cohort

**Figure 3:**
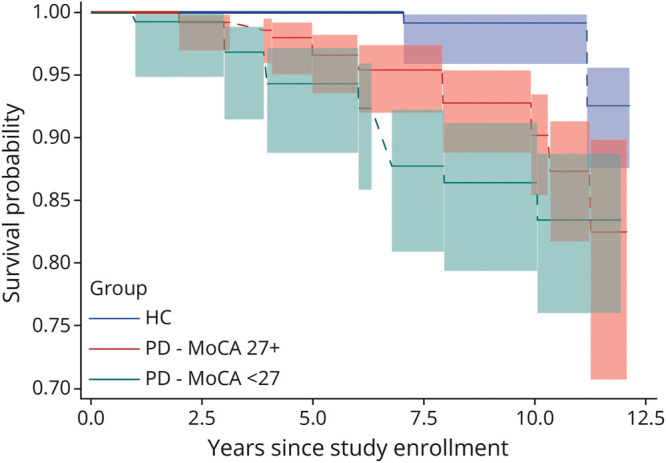
Time From Study Enrollment to Dementia Diagnosis for Participants With PD and HCs in the PPMI Cohort HC = healthy control; MoCA = Montreal Cognitive Assessment; PD = Parkinson disease; PPMI = Parkinson's Progression Markers Initiative.

Demographic characteristics of both groups are comparable and are presented in eTable 6. Of the 280 participants with PD and 192 HCs meeting the baseline MoCA criterion score ≥27, 266 participants with PD and 180 HCs had investigator-determined cognition diagnosis data, 19 (7.1%) and 2 (1.1%) of whom were diagnosed with dementia by an investigator over time (Figure 3). Among the 280 participants with PD and 192 HCs, 18 (6.4%) and 4 (2.1%) reached MoCA score <21 (eFigure 3) and 28 (10.0%) and 1 (0.5%) reached MDS-UPDRS item 1.1 score ≥3 (eFigure 4), respectively. Time to dementia from baseline by investigator diagnosis was significantly different between participants with PD and HCs (*p* = 0.002), as well as for proxy dementia definitions of MoCA <21 (*p* = 0.01) and MDS-UPDRS item 1.1 score ≥3 (*p* < 0.001). Figure 3 also demonstrates that participants with PD with a baseline MoCA score <27 progressed to dementia more rapidly than those with baseline MoCA scores ≥27.

### Worst-Case Sensitivity Analysis in PPMI Cohort

A total of 145 (36%) participants withdrew from PPMI before receiving a dementia diagnosis or study completion, including 137 by year 10. Under the worst-case assumption, 165 of 401 (41%) participants would be diagnosed with dementia within 10 years of PD diagnosis with a median time to dementia diagnosis including all time points of 11.4 years (95% CI 10.3–12.8). However, arguing against this assumption are the facts that the baseline visit characteristics did not significantly differ for participants who ultimately withdrew compared with those who did not (eTable 7) and that the cognitive end points obtained at the visit immediately before dropout suggested that these participants were cognitively intact on average at that time (eTable 8). So, while this is an unrealistic, and most extreme, assumption, it does provide a boundary of the estimate.

## Discussion

Analyzing data from two large, prospective, ongoing observational PD studies, we found that dementia may occur less frequently or develop over a longer period of time than has generally been assumed based on older studies. We also found that increasing age at disease diagnosis, male sex, and lower education level predicted development of dementia, consistent with previous literature.^36^

Combining the results of both cohorts, estimated risk of dementia by disease duration were 3%–12% at year 5, 9%–27% at year 10, 50% at year 15, 74% at year 20, and 90% from year 25 onward. Meanwhile, the aforementioned Norwegian study^15^ reported approximately 26% at year 9, 52% at year 13, and 78% at year 17 and the Australian study^16^ reported approximately 20% at year 5, 50% at year 10, 75% at year 15, and 83% at year 20. The mean time to dementia in the study by Hely et al. was 11 years in patients non-demented at baseline, compared with 15 years in the Penn cohort. The only other comparable cohorts, both following patients from disease onset, found the cumulative probability of dementia to be 46%,^17^ 54%,^19^ and 58%^18^ at approximately 10-year disease duration, with a median time to dementia diagnosis of 8.5 years in the latter. Thus, the Penn numbers are closer, although still lower at each time point, than what had been reported in previous comparable studies, while the PPMI numbers are significantly lower for the first 10 years of disease duration. In the Penn cohort at year 20, the mean age would be approximately 83 years, and the estimated lifetime risk of dementia in the general population is approximately 25%.^37^

When comparing participants with PD and HCs in the PPMI cohort with normal cognition at baseline (i.e., MoCA score ≥27), the PD group was significantly more likely to reach dementia, regardless of definition. Thus, although the PPMI cohort may be somewhat atypical in functioning and lifestyle compared with previously analyzed PD cohorts, the difference in dementia rates between participants with PD and HCs seen here is consistent with previous research studies.

Increasing age at disease diagnosis was associated with shorter time to dementia diagnosis, consistent with previous research showing a low long-term risk of dementia in patients with young-onset PD.^38^ This is not surprising because increasing age is also associated with an increased likelihood of comorbid Alzheimer disease and vascular pathology, which are also associated with cognitive impairment in PD.^39,40^ Findings on the effect of sex on dementia in PD has been mixed, but favors male patients being at increased risk,^36^ which we clearly observed. Those with less formal education also demonstrated faster progression to dementia in our cohort, again consistent with previous research.^36^

Strengths of analyses include that both studies are relatively large compared with previous studies, are current, have mean age at disease onset similar to other PD studies (i.e., 60–65 years), assess participants serially, administer both global and detailed cognitive assessments across multiple domains, and have a site investigator or consensus process to assign a cognitive diagnosis at each study visit. In addition, the PPMI study is multisite and international.

Limitations in the PPMI study were missing data in the outlying years (partially because of the coronavirus disease 2019 pandemic), although participants with PD with missing data had similar demographics and cognitive performance at the baseline visit to those who did not withdraw and at their last study visit had intact or only mild cognitive deficits. Other limitations for the PPMI study were reliance on the site investigator to diagnose dementia without requiring consideration of cognitive test results (although the MoCA cutoff provided similar results) and the site investigator diagnosis not being available for all participants until year three of the study. In comparing the participants with PD with HCs in the PPMI cohort, we are unable to say whether the two groups were drawn from the same population, although HCs were commonly spouses/partners of study participants, and the two groups were very similar to each other in age, sex, race, ethnicity, and education level. The Penn cohort had a longer duration between PD diagnosis and enrollment (0–32 years). It is possible that participants with advanced dementia were less likely to enroll in the study, which could result in an inflated estimated time to dementia diagnosis. Limitations for both cohorts include the facts that participants are highly educated and overwhelmingly White and were recruited specifically for participation in a research study. The atypical nature of the cohorts may help explain the low dementia risk in the PPMI cohort, but not differences in results between cohorts. We did not evaluate the impact of death as a competing risk, which could influence the estimated rates of dementia.

Differences in the long-term dementia estimates between cohorts were significant (9% vs 27% at year 10) and could be attributed to several factors. The PPMI cohort was specifically recruited for research participation close to the time of disease diagnosis and did not require treatment at study entry, whereas the Penn cohort was drawn from patients already receiving routine clinical care, and the cognitive consensus process at Penn may be more sensitive to assigning a dementia diagnosis because of its comprehensiveness. Future analyses could examine neurobiological predictors of conversion to dementia in the two cohorts, which may help further explain differences in dementia risk rates between the two cohorts.

Development of dementia is a long-term concern of patients with PD, and the combination of a motor and cognitive disorder can be devastating to patients and loved ones. This is increasingly recognized by clinicians, researchers, and those involved in treatment development. These results provide updated, and more hopeful, estimates of long-term dementia risk in PD, suggesting a longer window to intervene to prevent or delay cognitive decline.

## Appendix 1 Authors

**Table:**

Name	Location	Contribution
Julia Gallagher, BS	Department of Neurology, University of Pennsylvania, Philadelphia	Drafting/revision of the manuscript for content, including medical writing for content; major role in the acquisition of data; study concept or design; analysis or interpretation of data
Caroline Gochanour, MS	Department of Biostatistics, University of Iowa, Iowa City	Drafting/revision of the manuscript for content, including medical writing for content; analysis or interpretation of data
Chelsea Caspell-Garcia, MS	Department of Biostatistics, University of Iowa, Iowa City	Drafting/revision of the manuscript for content, including medical writing for content; analysis or interpretation of data
Roseanne D. Dobkin, PhD	Department of Psychiatry, Rutgers University, Newark, NJ	Drafting/revision of the manuscript for content, including medical writing for content
Dag Aarsland, MD, PhD	Department of Old Age Psychiatry, Kings College London, UK	Drafting/revision of the manuscript for content, including medical writing for content
Roy N. Alcalay, MD	Neurological Institute, Tel Aviv Sourasky Medical Center, Israel; Department of Neurology, Columbia University Irving Medical Center, New York, NY	Drafting/revision of the manuscript for content, including medical writing for content
Matthew J. Barrett, MD, MSc	Department of Neurology, Virginia Commonwealth University, Richmond	Drafting/revision of the manuscript for content, including medical writing for content
Lana Chahine, MD, MS	Department of Neurology, University of Pittsburgh, PA	Drafting/revision of the manuscript for content, including medical writing for content
Alice S. Chen-Plotkin, MD, MSc	Department of Neurology, University of Pennsylvania, Philadelphia	Drafting/revision of the manuscript for content, including medical writing for content
Christopher S. Coffey, PhD	Department of Biostatistics, University of Iowa, Iowa City	Drafting/revision of the manuscript for content, including medical writing for content
Nabila Dahodwala, MD, MS	Department of Neurology, University of Pennsylvania, Philadelphia	Drafting/revision of the manuscript for content, including medical writing for content
Jamie L. Eberling, PhD	The Michael J. Fox Foundation for Parkinson's Research, New York, NY	Drafting/revision of the manuscript for content, including medical writing for content
Alberto J. Espay, MD	James J. and Joan A. Gardner Family Center for Parkinson's Disease and Movement Disorders, Department of Neurology, University of Cincinnati, OH	Drafting/revision of the manuscript for content, including medical writing for content
James B. Leverenz, MD	Cleveland Clinic, Neurological Institute, Lou Ruvo Center for Brain Health, OH	Drafting/revision of the manuscript for content, including medical writing for content
Irene Litvan, MD	Department of Neuroscience, University of California San Diego	Drafting/revision of the manuscript for content, including medical writing for content
Eugenia Mamikonyan, MS	Department of Psychiatry, University of Pennsylvania, Philadelphia	Drafting/revision of the manuscript for content, including medical writing for content; major role in the acquisition of data; study concept or design; analysis or interpretation of data
James Morley, MD, PhD	Department of Neurology, University of Pennsylvania; Parkinson's Disease Research, Education and Clinical Center (PADRECC), Crescenz Veteran's Affairs Medical Center, Philadelphia	Drafting/revision of the manuscript for content, including medical writing for content
Irene H. Richard, MD	Department of Neurology, University of Rochester, NY	Drafting/revision of the manuscript for content, including medical writing for content
Liana Rosenthal, MD, PhD	Department of Neurology, Johns Hopkins University, Baltimore, MD	Drafting/revision of the manuscript for content, including medical writing for content
Andrew D. Siderowf, MD, MSCE	Department of Neurology, University of Pennsylvania, Philadelphia	Drafting/revision of the manuscript for content, including medical writing for content
Tatyana Simuni, MD	Department of Neurology, Northwestern University, Chicago, IL	Drafting/revision of the manuscript for content, including medical writing for content
Michele K. York, PhD	Departments of Neurology and Psychiatry and Behavioral Sciences, Baylor College of Medicine, Houston, TX	Drafting/revision of the manuscript for content, including medical writing for content
Allison W. Willis, MD, MSCI	Department of Neurology, University of Pennsylvania, Philadelphia	Drafting/revision of the manuscript for content, including medical writing for content
Sharon X. Xie, PhD	Department of Biostatistics and Epidemiology, University of Pennsylvania, Philadelphia	Drafting/revision of the manuscript for content, including medical writing for content
Daniel Weintraub, MD	Department of Neurology, and Department of Psychiatry, University of Pennsylvania; Parkinson's Disease Research, Education and Clinical Center (PADRECC), Crescenz Veteran's Affairs Medical Center, Philadelphia, PA	Drafting/revision of the manuscript for content, including medical writing for content; major role in the acquisition of data; study concept or design; analysis or interpretation of data

## Appendix 2 Coinvestigators

**Table:**

Coinvestigators are listed online at Neurology.org.

## Glossary

HC: healthy control
MCI: mild cognitive impairment
MDS-UPDRS: Movement Disorder Society—Unified Parkinson's Disease Rating Scale
MoCA: Montreal Cognitive Assessment
PD: Parkinson disease
PDD: PD dementia
Penn: University of Pennsylvania
PPMI: Parkinson's Progression Markers Initiative
